# Alpha‐Asarone modulates kynurenine disposal in muscle and mediates resilience to stress‐induced depression via PGC‐1α induction

**DOI:** 10.1111/cns.14030

**Published:** 2022-12-27

**Authors:** Lu Yan, Chu‐han Liu, Li Xu, Yi‐yun Qian, Ping‐ping Song, Min Wei, Bao‐lin Liu

**Affiliations:** ^1^ Jiangsu Key Laboratory for the Research and Utilization of Plant Resources Institute of Botany, Jiangsu Province and Chinese Academy of Sciences Nanjing China; ^2^ Affiliated Mental Health Center & Hangzhou Seventh People's Hospital Zhejiang University School of Medicine Hangzhou China; ^3^ State Key Laboratory of Environmental Chemistry and Ecotoxicology Research Center for Eco‐Environmental Science, Chinese Academy Sciences Beijing China; ^4^ State Key Laboratory of Natural Medicines, School of Traditional Chinese Pharmacy China Pharmaceutical University Nanjing China

**Keywords:** depression, kynurenine metabolism, PGC‐1α, α‐asarone

## Abstract

**Introduction:**

Kynurenine (KYN) accumulation in periphery induces brain injury, responsible for depression. α‐Asarone is a simple phenylpropanoids that exerts beneficial effects on central nervous system. However, the effect of α‐asarone on periphery is unexplored.

**Aims:**

Here, we investigated its protective role against depression from the aspect of KYN metabolism in skeletal muscle.

**Methods:**

The antidepressant effects of α‐asarone were evaluated in chronic mild stress (CMS) and muscle‐specific PGC‐1α‐deficient mice. The effects of KYN metabolism were determined in mice and C2C12 myoblasts.

**Results:**

α‐Asarone exerted antidepressant effects in CMS and KYN‐challenged mice via modulating KYN metabolism. In myoblasts, α‐asarone regulated PGC‐1α induction via cAMP/CREB signaling and upregulated KYN aminotransferases (KATs) to increase KYN clearance in a manner dependent on PGC‐1α. KAT function is coupled with malate–aspartate shuttle (MAS), while α‐asarone combated oxidative stress to protect MAS and mitochondrial integrity by raising the NAD^+^/NADH ratio, ensuring effective KYN disposal. In support, the antidepressant effect of α‐asarone was diminished by muscle‐specific PGC‐1α deficient mice subjected to KYN challenge.

**Conclusion:**

KATs coupled with MAS to clear KYN in muscle. α‐Asarone increased PGC‐1α induction and promoted KYN disposal in muscle, suggesting that protection of mitochondria is a way for pharmacological intervention to depression.

## Graphical abstract

α‐Asarone modulates PGC‐1α induction via cAMP/CREB signaling and combats oxidative stress to protect MAS and mitochondrial integrity by raising the NAD^+^/NADH ratio to improve KAT/MAS integration, and thereby facilitating KYN clearance in muscle and mediating resilience to depression.

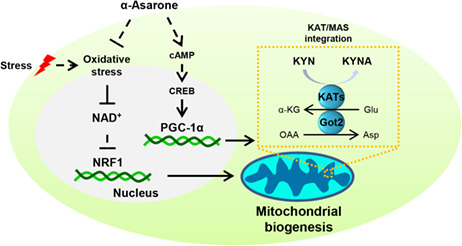

## 1 INTRODUCTION

Depression is a heterogeneous disorder characterized by impaired mood and reduced physical functioning. It is generally accepted that disturbances in glutamate transmission and synaptic plasticity are the main causes of depression.^1^, ^2^, ^3^ However, emerging evidence reveals the potential impact of peripheral disorders on neuronal function. Alteration in gut microbiota richness and diversity is associated with depression and the findings from experimental studies and clinical trials also show the coexistence of peripheral inflammatory diseases with depression.^4^, ^5^, ^6^ Amino acid metabolism is contributable to the synthesis of neurotransmitters. Although tryptophan is mainly degraded in peripheral tissues, its kynurenine (KYN) metabolite is neurotoxic,^7^, ^8^ which can cross the blood–brain barrier (BBB). These events address that peripheral modulation might be an important way to alleviate depression.

Tryptophan is the precursor of serotonin synthesis, while the KYN pathway is the major catabolic route of tryptophan and over 90% of peripheral tryptophan is metabolized into KYN and downstream neuroactive metabolites.^9^ The conversion of tryptophan to KYN is enabled by tryptophan 2,3‐dioxygenase (TDO) and indoleamine 2,3‐dioxygenase (IDO). Chronic stress could activate IDO and TDO to increase KYN accumulation in the brain,^10^ responsible for neuropathological changes.^11^, ^12^ Skeletal muscle presents high levels of KYN aminotransferases (KATs) which promote the conversion of KYN to kynurenic acid (KYNA) to reduce neurotoxicity. As opposed to KYN, KYNA is unable to cross BBB.^13^, ^14^ Therefore, promoting KYN disposal in the muscle can prevent KYN accumulation and neurotoxicity. The malate–aspartate shuttle (MAS) acts to transfer reducing equivalents from nicotinamide adenine dinucleotide (NADH) in the cytosol to the mitochondria for the renewal of NAD^+^ to maintain redox homeostasis, and KATs are involved in the reactions of MAS, through which transferring the amino group from KYN to generate glutamate and aspartate to support metabolite pools. The KAT/MAS integration increases KYN disposal and energy efficiency to maintain the function of skeletal muscle, supported by mitochondrial function.^15^ Peroxisome proliferator‐activated receptor‐γ coactivator‐1α (PGC‐1α) is a transcriptional co‐regulator that regulates mitochondrial biogenesis and enhances oxidative phosphorylation.^16^ Skeletal muscle PGC‐1α promoted KYN disposal to protect against stress‐induced depression,^13^ indicative of the functional interaction between muscle PGC‐1α and neuroprotection.

Acori Tatarinowii Rhizoma (Shichangpu in Chinese) is the dried rhizome of *Acorus tatarinowii* Schott and has been used in traditional Chinese medicine for the treatment of neurological disorders.^17^ α‐Asarone is a simple phenylpropanoids in Acori Tatarinowii Rhizoma, exerting beneficial effects on alleviating neuronal excitotoxicity, anti‐inflammation and neurotransmission, and these studies are mainly focused on the regulation of central nervous system.^18^, ^19^, ^20^ However, the role of α‐asarone in peripheral regulation against depression remains unclear. In the present study, we investigated the antidepressant effect of α‐asarone in mouse model from the aspect of KYN metabolism in skeletal muscle. We demonstrated that the coupling with MAS was required for KYN metabolism in muscle, susceptible to stress damage. α‐Asarone regulated PGC‐1α and protected mitochondria from oxidative damage to promote KYN disposal in muscle.

## 2 MATERIALS AND METHODS

### 2.1 Materials

α‐Asarone (purity >95%) was purchased from Weikeqi‐Biotech Co., Ltd. ZLN005 (HY‐17538), 666‐15 (HY‐101120), β‐nicotinamide mononucleotide (NMN, HY‐F0004) and aminooxyacetic acid (AOA, HY‐107994) were from MCE China. KYN (K3750), forskolin (F3917), H89 (B1427) and other reagents were from Sigma‐Aldrich.

### 2.2 Animal experiments

Male C57BL/6 mice (8‐ to 10‐week‐old at the start of experiments), weighing 18–22 g, were obtained from Changzhou Cavens Laboratory Animal Co. Ltd. (Changzhou, China). Animals were hosted on a 12 h light/dark cycle (lights on at 6:00 a.m. and off at 6:00 p.m.) under controlled temperature (22 ± 2°C) and humidity (50% ± 10%), with standard diet and water ad libitum. Animals were acclimatized for 7 days. All animal care and experiments were carried out in accordance with the National Institutes of Health guide for the care and use of Laboratory animals (NIH Publication No. 85‐23, revised 2011). All animal studies complied with the ARRIVE guidelines. Animal Experimentation Ethics Committee of China Pharmaceutical University, where appropriate, authorized the project license. All efforts were made to minimize suffering.

Sucrose adaptation and sucrose consumption assessment were carried out before the chronic mild stress (CMS) procedures at the beginning of the experiment. The procedures of CMS were conducted with the published study with some adjustments.^13^ Briefly, a series of stressors were applied onto the animals: (1) stroboscopic illumination for 2 h, (2) tilted cage for 16 h, (3) loud noise for 2 h, (4) wet cage for 15 h, (5) restraint for 1–2 h, (6) food restriction for 6 h, and (7) day and night reverse. These stressors were randomly arranged in 1 week and repeated for 6 weeks. Mice were randomized and orally administrated with saline or α‐asarone (5, 15 mg/kg) for 6 weeks. The body weight of all mice was recorded every week.

In acute KYN challenge test, mice were orally administrated with saline or α‐asarone (15 mg/kg) for 1 week and then 2.5 mg/kg of KYN or saline were injected intraperitoneally for 4 h.

For the muscle‐specific PGC‐1α knockdown in mice, AAV9‐sh*Ppargc1α* and AAV9‐shNC (negative control) viruses were designed by Genomeditech (Shanghai, China). After anesthesia, male C57BL/6 mice were injected with 100 μl of AAV9‐sh*Ppargc1α* at a concentration of 1E12 viral genomes (vg)/ml or AAV9‐shNC at a concentration of 1E12 vg/ml through the gastrocnemius injection. 4 weeks later, muscle *Ppargc1α* mRNA expression was examined to confirm the efficiency of knockdown. The target sequence of AAV9‐sh*Ppargc1α* is 5′‐GCAACATGCTCA AGCCAAACC‐3′, and the sequence of AAV9‐shNC is 5′‐TTCTCCGAACGTGTC ACGT‐3′. Then, the mice were orally administrated with saline or α‐asarone (15 mg/kg) for 1 week followed by 2.5 mg/kg of KYN or saline via intraperitoneal injection. Sucrose consumption was assessed 4 h after injection.

### 2.3 Behavior tests

Sucrose preference test was carried out at the end of CMS procedures and 4 h after KYN challenge. In brief, mice in each group were learned to adapt to 2 bottles of 1% sucrose solution (w/v) 72 h before the test, and 24 h later, one bottle of 1% sucrose solution (w/v) was replaced with tap water for 24 h. Then, mice were deprived of water and food for 24 h. Sucrose preference test was conducted at 17:00 p.m., where mice were kept in individual cages with 2 bottles, one with 100 ml of 1% sucrose solution (w/v) and the other with 100 ml of water. After 3 h, the volumes of consumed sucrose solution and water were recorded.^21^

Forced swimming test was carried out at the end of CMS procedures. Mice in each group were placed in large glass cylinders (50 cm height and 20 cm diameter) with 30 cm height water at 22 ± 2°C, so that mice were not able to support themselves by hind limbs. The test consisted of two parts: the first 15 min was for preswimming and then 24 h later, the swimming behavior was observed in 5 min, and the latency to float was measured and analyzed.^22^

### 2.4 Cell culture and transfection

C2C12 cells were obtained from ATCC (Manassas, VA, ATCC Cat# CRL‐1772). C2C12 myoblasts and siRNA interference have been previously described.^23^ Fully differentiated C2C12 myoblasts were treated with α‐asarone at given concentrations before 10 μM KYN for 4 h, or treated with 1 μM 666‐15 (CREB inhibitor), 5 μM H89 (PKA inhibitor), or 100 μM AOA (MAS inhibitor) 3 h before 10 μM α‐asarone for 12 h and 10 μM KYN for 4 h. The cells were transfected with siRNA for *Ppargc1α* (Sangon Biotech, R6187, CCUCCUCAUAAAGCCAACCAATT, UUGGUUGGCUUUAUGAGGAGGTT, shanghai, China), *Nrf1* (Sangon Biotech, R9768, CCCGAGGACACUUCUUAUGAUTT, AUCAUAAGAAGUGUCCUCGGGTT), *Slc25a11* (Sangon Biotech, R9768, CGGAUGCAGUUGAGUGGUGAATT, UUCACCACUCAACUGCAUCCGTT), *Slc25a12* (Sangon Biotech, R9768, CGCAUUUAACUCCUUACUCAATT, UUGAGUAAGGAGUUAAAUGCGTT), or NC (UUCUCCGAACGUGUCACGUTT, ACGUGACACGUUCGGAGAATT) using Lipofectamine® 2000 transfection reagent (Thermo Fisher Scientific, 11668019) and then differentiated.

### 2.5 Real‐time quantitative PCR

Total RNA was isolated from mouse hippocampus, gastrocnemius muscle, or cell samples by RNAprep pure Tissue Kit (Tiangen) according to the manufacturer's instructions. The concentrations of RNAs were detected by UV absorbance at 260 nm. cDNA was reverse transcribed from 1 μg samples of total RNA using RT SuperMix for qPCR (Vazyme). Real‐time PCR was performed using SYBR Green Master Mix (Vazyme). The SYBR green signal was detected by qTOWER 2.0 (Analytic Jena AG). Primer sequences are listed in Table S1.

### 2.6 Kit measurements

NAD^+^/NADH quantification from gastrocnemius muscle and cell samples were done using commercially available kits (Abcam, ab65348, Cambridge, MA; AAT Bioquest, 15273, Sunnyvale, CA), following manufacturer's instruction. Activities of adenylyl cyclase (AC) and poly(ADP‐ribose) polymerase‐1 (PARP1) and levels of cyclic adenosine monophosphate (cAMP), KYN, 3‐hydroxykynurenine (3‐HK), KYNA, ROS, 8‐hydroxy‐2′‐deoxyguanosine (8‐OHdG), from gastrocnemius muscle and cell samples, were conducted using commercially available kits (Lanpaibio, LP‐M02586, LP‐M06254, LP‐M03797, LP‐M06202, LP‐M03219, LP‐M06205, LP‐M01005, LP‐M05277, Shanghai, China).

### 2.7 Transmission electron microscopy and image analyses

To observe the morphology of mitochondria in gastrocnemius muscle, the fresh muscle was rapidly fixed with electron microscope fixation solution at 4°C for 2–4 h. After washing with 0.1 M PBS, the muscle was fixed with 1% osmic acid at room temperature for 2 h in darkness. After gradient dehydration, the tissue was embedded and sliced, followed by staining with 2% uranyl acetate and 2.6% lead citrate for 8 min, respectively. Transmission electron microscopy (HITACHI, HT7700) was used for image acquisition and analysis. Mitochondria were manually traced in nonoverlapping electron micrograph sections of 100 μm^2^. Mitochondria number and area were quantified with ImageJ ROI manager (NIH). Mean area was obtained as the mean surface of individual mitochondria in a given micrograph.

### 2.8 SDS‐PAGE and immunoblotting

The cultured cells were collected, and protein content was determined by Bradford method. Proteins (~20 μg) were separated on 8% SDS‐polyacrylamide gels and transferred to a PVDF membrane. The PVDF membrane was blocked with 5% fat‐free milk in tris‐buffer saline/0.1% tween 20 (TBS‐T) and then incubated in the primary antibodies diluted in 2.5% fat‐free milk in TBS‐T over night at 4°C. The primary antibodies were anti‐phospho‐CREB (1:1000, Cell Signaling Technology, 9198, Cat# ABIN461313), anti‐CREB (1:1000, Cell Signaling Technology, 9197, Cat# 27‐321), anti‐PGC‐1α (1:1000, Sangon Biotech, D162041, Cat# sc‐518025), anti‐β‐actin (1:5000, Sangon Biotech, D110001, Cat# 130‐120‐277), and anti‐GAPDH (1:5000, Sangon Biotech, D110016, Cat# JM‐3777‐100). After that, the PVDF membrane was rinsed with TBS‐T and incubated for 2 h at room temperature in peroxidase (HRP)‐conjugated anti‐rabbit secondary antibody (1:5000, Sangon Biotech, D110058), diluted in 2.5% fat‐free milk in TBS‐T. After intensive washing with TBS‐T, the immune complexes were visualized using the enhanced chemiluminescence (ECL) method (Vazyme). The intensities of bands in control and samples, run on the same gel and under strictly standardized ECL conditions, were compared on an image analyzer, using a calibration plot constructed from a parallel gel with serial dilutions of one of the sample.

### 2.9 Luciferase reporter assay

The *Ppargc1α* promoter (~1200 bps relative to the TSS) were synthesized and inserted in the pGL3‐basic vector between Nhel and HindIII sites (Genebay Biotech). Combined with the plasmid, additional pRL‐SV40‐Renilla vector (as a normalized control) was cotransfected into C2C12 myoblasts in the presence or absence of pReceiver‐M02 encoding CREB using Lipofectamine® 2000 transfection reagent (Thermo Fisher Scientific) at 70%–80% confluence before differentiation. 48 hours after transfection, the cells were treated with indicated reagents and then collected. The activities of firefly and renilla luciferase were detected by the dual‐luciferase substrate system (Genecopia, LF004) according to the manufacturer's instruction.

### 2.10 Mitochondrial mass assay

For the analysis of mitochondrial mass, the treated C2C12 myoblasts were cultured with 10 μM NAO (Thermo Fisher Scientific, A1372) at 37°C for 30 min in darkness. After washing with PBS, the cells were observed by confocal scanning microscopy (Zeiss, LSM 900). According to the manufacturer's instruction, cellular fluorescence was imaged using confocal scanning microscopy.

### 2.11 Statistical analysis

Statistical analysis was performed using GraphPad Prism. Shapiro–Wilk test was applied to check the normal distribution of the data, while *F*‐test was used to check homogeneity of variances. Data were presented as mean ± SEM (*n* ≥ 5) and were compared between groups using two‐tailed *t*‐test or one‐way ANOVA followed by Tukey's test when normally distributed. Mann–Whitney *U* and Kruskal‐Wilcoxon tests were performed if the data were not normally distributed. *p* < 0.05 was considered statistically significant.

## 3 RESULTS

### 3.1 α‐Asarone alleviates chronic mild stress‐induced depression in mice

**FIGURE 1:**
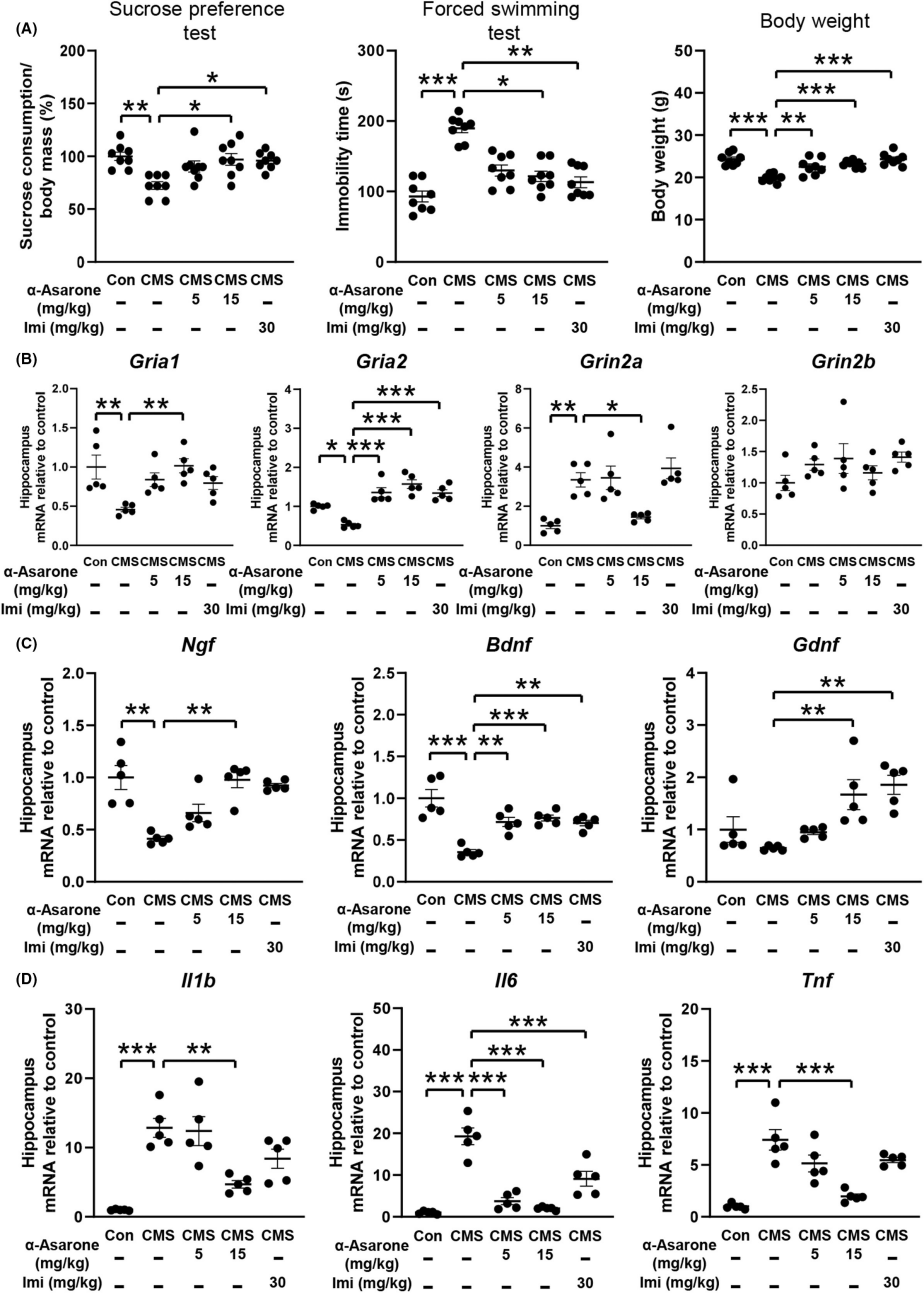
α‐Asarone alleviates CMS‐induced depression in mice. (A) Sucrose preference test, forced swimming test, and body weight (*n* = 8). (B) Gene expression of AMPA receptor subunits and NMDA receptor subunits in hippocampus (*n* = 5). (C) Gene expression of neurotrophic factors in hippocampus (*n* = 5). (D) Gene expression of proinflammatory cytokines in hippocampus (*n* = 5) (chronic mild stress, CMS; imipramine, Imi). Data are expressed as mean ± SEM, *p < 0.05, **p < 0.01, ***p < 0.001 compared with CMS.

To explore the role of α‐asarone against depression, a depression mouse model with CMS was established as it closely mimics the situation to clinical depression.^24^ After 6 weeks of exposure to CMS, mice developed depressive behaviors, as indicated by decreased sucrose consumption and increased immobility time in forced swimming test, accompanied with weight loss. Oral administration of α‐asarone (5, 15 mg/kg) effectively alleviated depressive behaviors (Figure 1A), while the behaviors in normal mice were not affected (Figure S1). Imipramine (30 mg/kg) is a tricyclic antidepressant used here as a positive control.

Depression is characterized by abnormality in glutamate transmission and synaptic plasticity in the brain.^25^, ^26^ CMS impaired hippocampal gene induction of AMPA receptor subunits *Gria1* and *Gria2*, but increased the expression of NMDA receptor subunit *Grin2a*, with *Grin2b* remained unchanged, whereas these alterations were reversed by α‐asarone (Figure 1B), indicative of the ability to improve synaptic plasticity. Deficiency of neurotrophic factors is proposed to contribute to depression,^27^, ^28^ and α‐asarone increased hippocampal mRNA levels of neurotrophic factors (*Ngf*, *Bdnf*, and *Gdnf*) in dose‐dependent manners (Figure 1C). Proinflammatory cytokines are important promoting factors in the pathogenesis of stress‐induced depression.^29^, ^30^ Long‐term exposure to chronic stress caused gene induction of proinflammatory cytokines (*Il1b*, *Il6*, and *Tnf*) that were suppressed dose‐dependently by α‐asarone (Figure 1D). Collectively, these results demonstrated that α‐asarone effectively prevented hippocampal abnormality, rendering resistance to depressive symptoms.

### 3.2 α‐Asarone promotes kynurenine disposal in muscle

**FIGURE 2:**
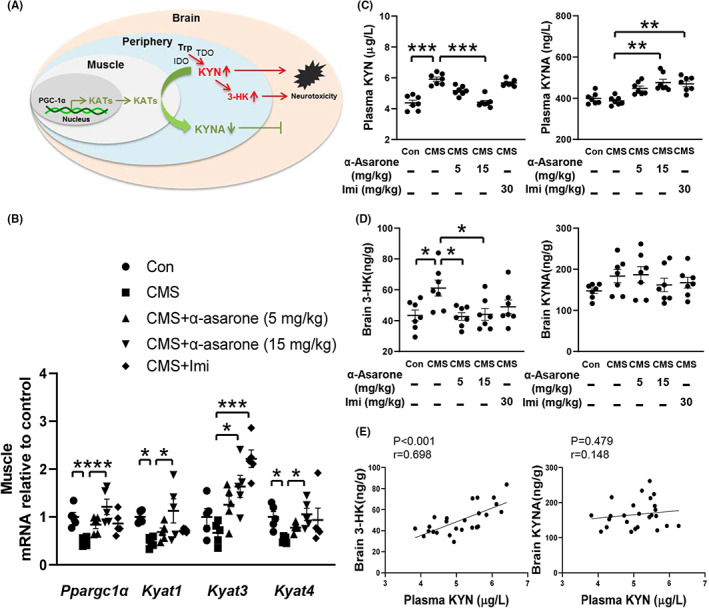
α‐Asarone regulates KYN metabolism in skeletal muscle. (A) KYN metabolism in skeletal muscle. (B) Gene expression of *Ppargc1α*, *Kyat1*, *Kyat3*, and *Kyat4* in gastrocnemius muscle (*n* = 5). (C) Plasma concentrations of KYN and KYNA (*n* = 7). (D) 3‐HK and KYNA concentrations in brain tissue (*n* = 7). (E) Correlation between plasma KYN and brain 3‐HK and KYNA levels with each circle representing an individual animal (chronic mild stress, CMS; imipramine, Imi). Data are expressed as mean ± SEM, *p < 0.05, **p < 0.01, ***p < 0.001 compared with CMS.

In response to stress or proinflammatory factors, the KYN pathway of tryptophan degradation is activated which accounts for the majority of peripheral tryptophan metabolism.^31^ By transporting across the BBB, KYN is degraded into 3‐HK, both are neurotoxic (Figure 2A). When CMS impaired KYN metabolism in skeletal muscle, α‐asarone concentration‐dependently restored *Ppargc1α* expression with upregulation of *Kyat1*, *Kyat3*, and *Kyat4*, which encodes KATs to mediate the conversion of KYN to KYNA (Figure 2B). α‐Asarone reduced circulating KYN contents with a corresponding increase in KYNA contents in a concentration‐dependent effect, indicating that it promoted the conversion of KYN to KYNA in peripheral tissues (Figure 2C). As expected, α‐asarone reduced 3‐HK contents in the brain, largely due to limited KYN entering the brain (Figure 2D). In support of this, plasma KYN levels correlated with brain 3‐HK contents but not with KYNA (Figure 2E). Despite the alterations in KYN metabolism, the levels of tryptophan or serotonin in the blood were not influenced under any treatment (Figure S2).

### 3.3 α‐Asarone protects muscle KATs against KYN insult

**FIGURE 3:**
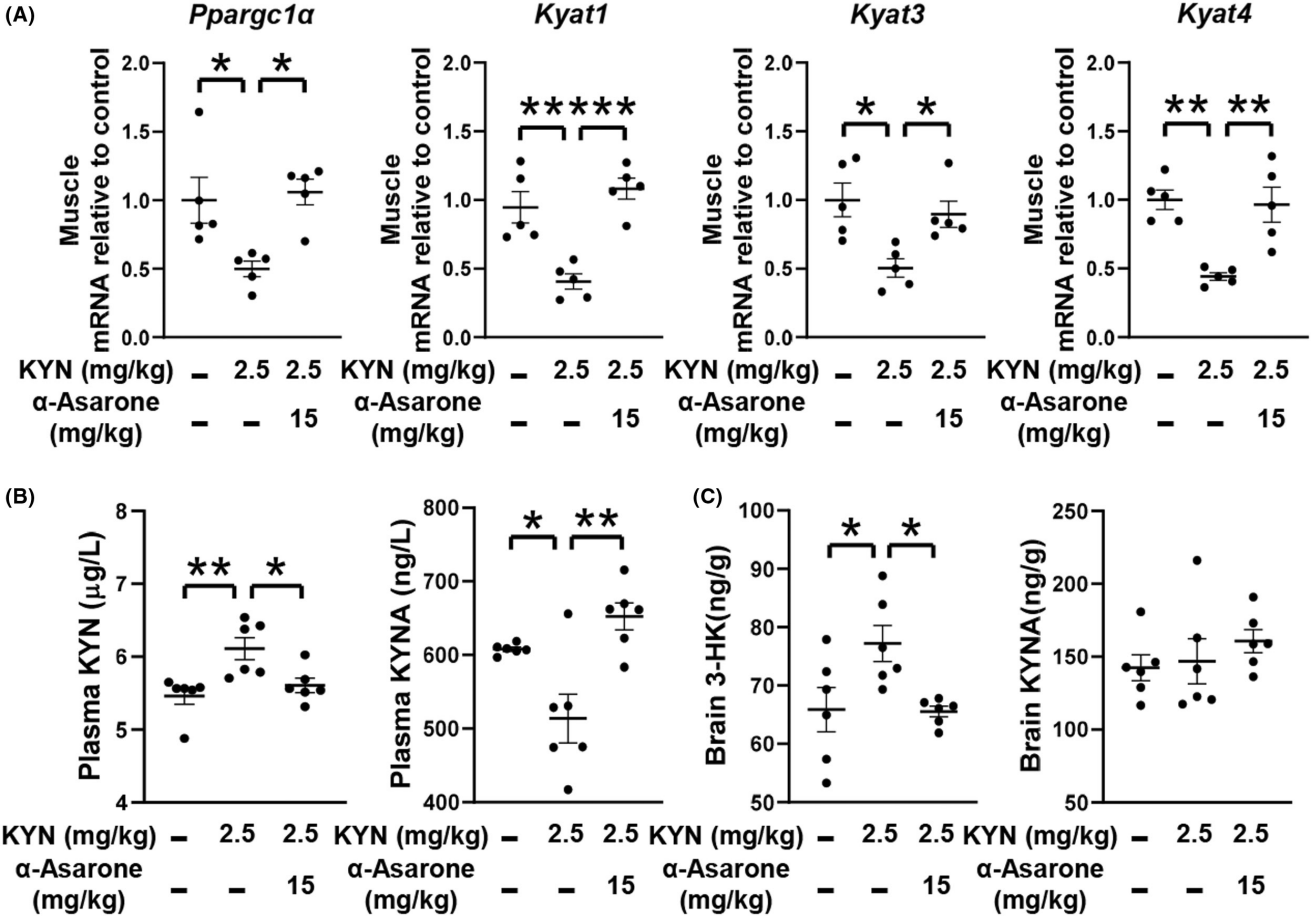
α‐Asarone promotes muscle KYN disposal against KYN insult. (A) Gene expression of *Ppargc1α*, *Kyat1*, *Kyat3*, and *Kyat4* in gastrocnemius muscle (*n* = 5). (B) Plasma concentrations of KYN and KYNA (*n* = 6). (C) 3‐HK and KYNA concentrations in brain tissue (*n* = 6). Data are expressed as mean ± SEM, *p < 0.05, **p < 0.01, ***p < 0.001 compared with KYN.

To mimic KYN accumulation responding to stress, we treated mice with intraperitoneal injection of KYN to observe the impact on skeletal muscle. KYN administration induced depressive‐like behavior in mice, as reported in previous study.^13^ KYN challenge impaired gene induction of *Ppargc1α*, *Kyat1*, *Kyat3*, and *Kyat4* in the muscle, whereas oral administration of α‐asarone reversed these alternations (Figure 3A). As expected, α‐asarone reduced the elevated levels of circulating KYN with a corresponding increase in KYNA contents (Figure 3B). α‐Asarone reduced 3‐HK accumulation in the brain, despite no significant influence on KYNA level (Figure 3C). While peripheral KYN, but not KYNA, is capable of entering the brain, where it is degraded to 3‐HK by resident microglia and macrophage.^8^

### 3.4 α‐Asarone regulates PGC‐1α via cAMP/CREB signaling cascades

**FIGURE 4:**
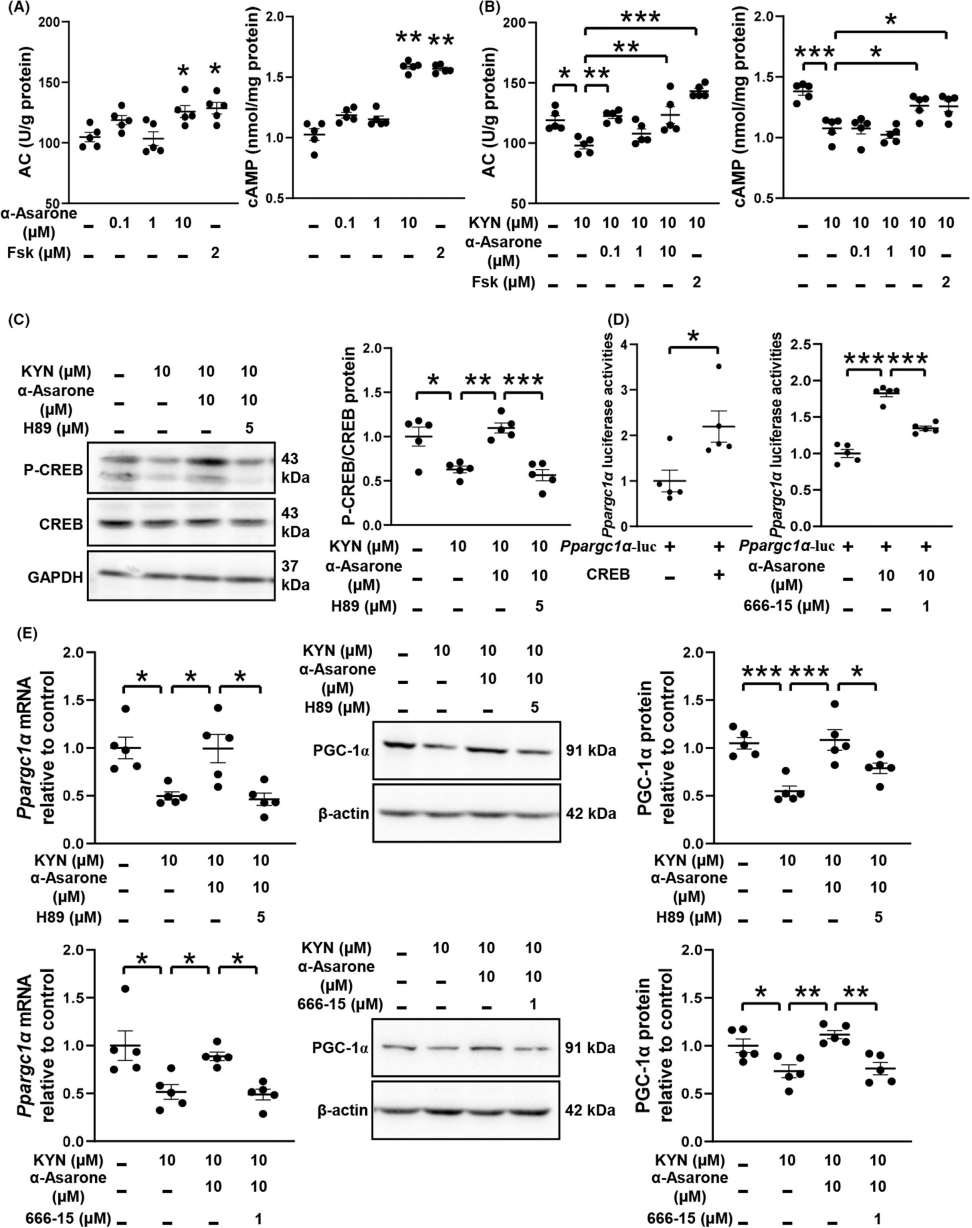
α‐Asarone regulates PGC‐1α induction in myoblasts. (A, B) AC activity and cAMP contents in myoblasts. (C) P‐CREB/CREB protein abundance in myoblasts. (D) *Ppargc1α* promoter activity in myoblasts. (E) Gene and protein expression of PGC‐1α in myoblasts. (foskolin, Fsk). Data are expressed as mean ± SEM, where *n* = 5, *p < 0.05, **p < 0.01, ***p < 0.001.

PGC‐1α is responsible for the transcriptional regulation of KATs in KYN metabolism.^32^ Although a number of activators of PGC‐1α have been found, the most potent activator is CREB.^33^ In response to cAMP, PKA activates CREB by phosphorylation. The selected dose of α‐asarone in C2C12 myoblasts was determined by MTT assays, i.e. the dose did not affect the cell number as shown in Figure S3. In C2C12 myoblasts, α‐asarone increased AC activity and cAMP generation (Figure 4A). Similar regulation was also observed when exposed to KYN (Figure 4B). KYN also reduced CREB phosphorylation in myoblasts which was rescued by α‐asarone, while the protective effect was blocked by PKA inhibitor H89 (Figure 4C). Luciferase reporter showed that CREB overexpression increased *Ppargc1α* promoter activity, and the role of α‐asarone in upregulation of *Ppargc1α* promoter activity was blocked by CREB inhibitor 666‐15 (Figure 4D). α‐Asarone protected PGC‐1α gene expression and protein abundance, whereas these effects were diminished by PKA inhibitor H89 and CREB inhibitor 666‐15, respectively (Figure 4E). Collectively, these results indicated that α‐asarone regulates PGC‐1α induction via cAMP/CREB signaling cascades.

### 3.5 α‐Asarone regulates KATs dependently on PGC‐1α

**FIGURE 5:**
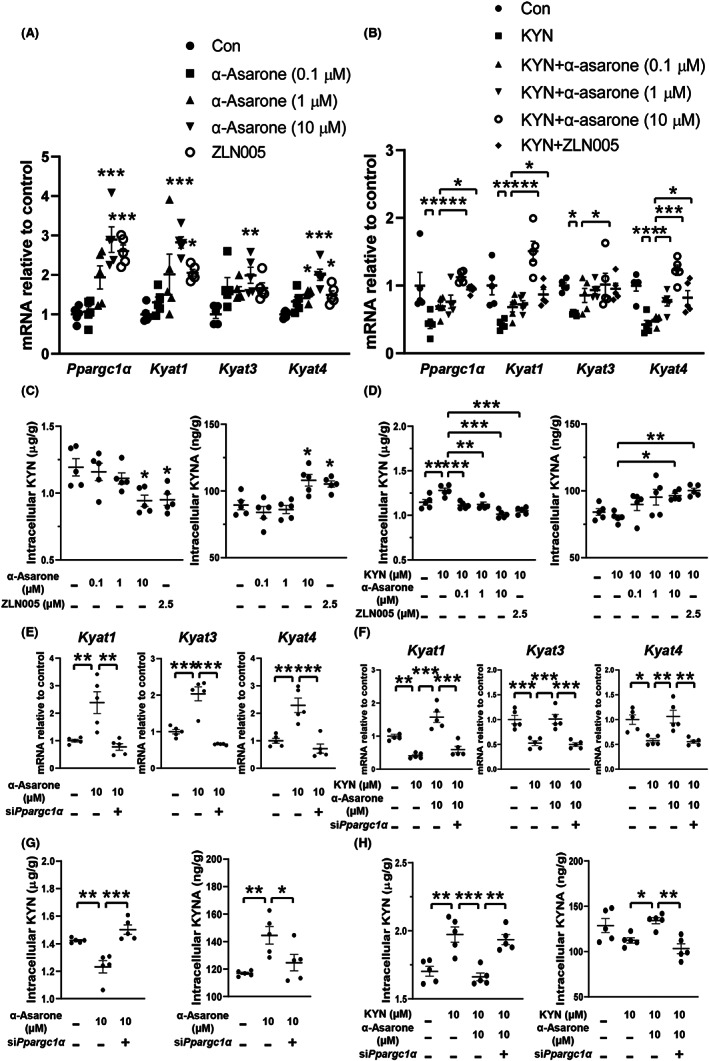
α‐Asarone regulates KATs dependently on PGC‐1α induction. (A, B) Gene expression of *Ppargc1α*, *Kyat1*, *Kyat3*, and *Kyat4* in myoblasts. (C, D) KYN and KYNA levels in myoblasts. (E, F) Gene expression of *Kyat1*, *Kyat3*, and *Kyat4* in myoblasts with si*Ppargc1α*. (G, H) KYN and KYNA levels in myoblasts with si*Ppargc1α*. (KYN, 10 μM; ZLN005, 2.5 μM). Data are expressed as mean ± SEM, where *n* = 5, *p < 0.05, **p < 0.01, ***p < 0.001.

We then investigated the regulation of KYN metabolism in C2C12 myoblasts. Similar to PGC‐1α activator ZLN005, α‐asarone concentration‐dependently increased gene expression of *Ppargc1α*, *Kyat1*, *Kyat3*, and *Kyat4* (Figure 5A). When *Ppargc1α* was silenced using siRNA, the upregulation of *Kyat1*, *Kyat3*, and *Kyat4* mRNA expression by α‐asarone in untreated cells was diminished (Figure 5E). Moreover, α‐asarone protected these gene expressions against KYN insult in a manner dependent on *Ppargc1α* (Figure 5B,F). These results showed that PGC‐1α induction was required for α‐asarone to regulate KAT expression. Consistently, α‐asarone treatment reduced KYN levels with a parallel increase in KYNA production in both untreated and KYN‐stimulated muscle cells (Figure 5C,D). In myoblasts transfected with si*Ppargc1α*, the conversion of KYN to KYNA by α‐asarone was eliminated (Figure 5G,H), providing evidence that α‐asarone shifted KYN metabolism toward KYNA by PGC‐1α induction in muscle cells.

### 3.6 α‐Asarone preserves NAD ^+^ to improve KAT/MAS integration against KYN insult

**FIGURE 6:**
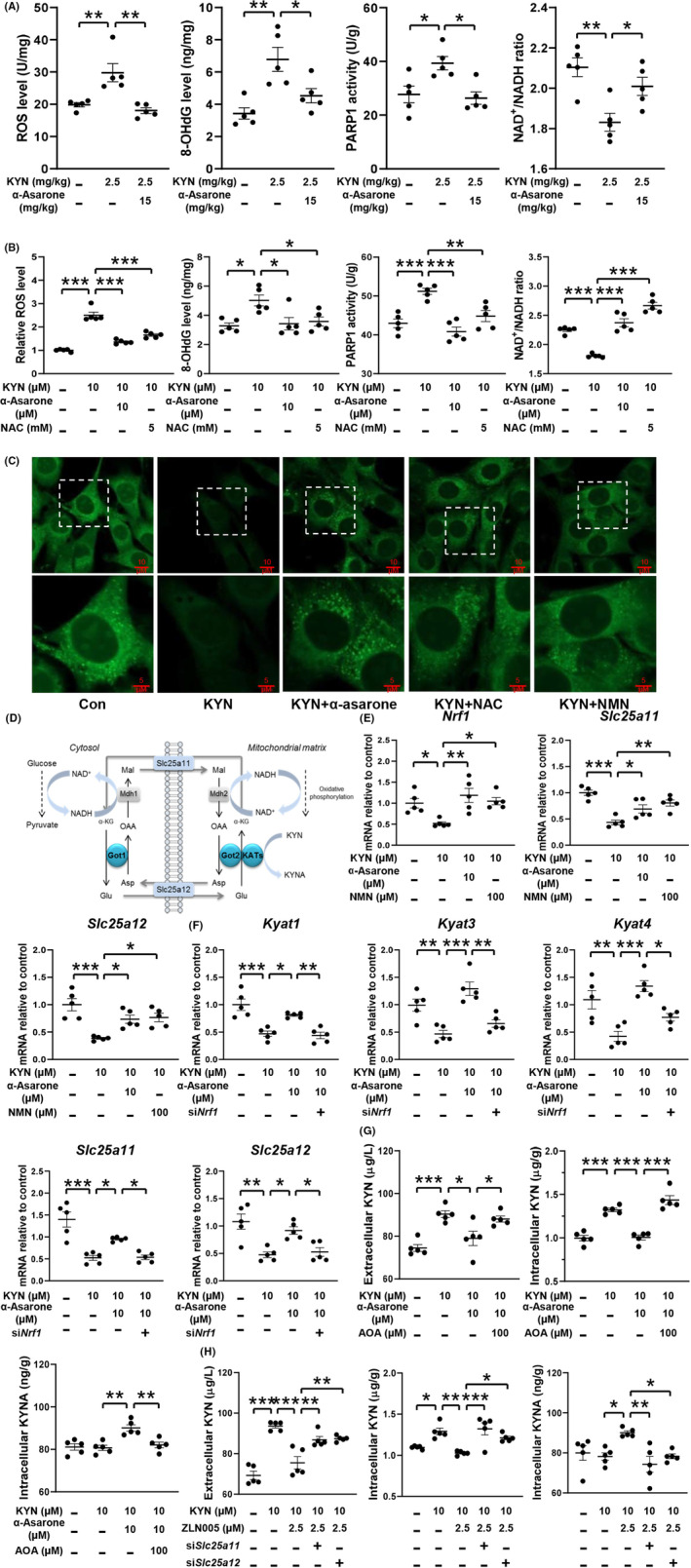
α‐Asarone improves KAT/MAS integration against KYN insult. (A) ROS and 8‐OHdG levels, PARP1 activity, NAD^+^/NADH ratio in gastrocnemius muscle. (B) ROS and 8‐OHdG levels, PARP1 activity, NAD^+^/NADH ratio in myoblasts. (C) Mitochondrial mass in myoblasts staining with NAO (10 μM), scale bar = 10 μm or 5 μm. (D) Schematic representation of MAS. (E) Gene expression involved in MAS. (F) Gene expression in myoblasts with si*Nrf1*. (G) KYN and KYNA levels in myoblasts in the presence of AOA (100 μM). (H) KYN and KYNA levels in myoblasts with si*Slc25a11* or si*Slc25a12*. (KYN, 10 μM; NAC, 5 mM; NMN, 100 μM; N‐acetyl‐L‐cysteine, NAC; β‐nicotinamide mononucleotide, NMN; aminooxyacetic acid, AOA). Data are expressed as mean ± SEM, where *n* = 5, *p < 0.05, **p < 0.01, ***p < 0.001 compared with KYN.

KYN challenge increased ROS generation and 8‐OHdG contents in mouse muscle, but the oxidative damage was prevented by α‐asarone (Figure 6A). In response to DNA impairment, PARP1 repairs impaired DNA at the expense of NAD^+^ consumption.^34^, ^35^ α‐Asarone inactivated PARP1 and thus increased the ratio of NAD^+^/NADH in KYN‐treated mouse muscle (Figure 6A). Similar to the regulation in vivo, α‐asarone, as well as ROS scavenger NAC, suppressed ROS production, reduced 8‐OHdG accumulation, and inactivated PARP1 with normalized NAD^+^/NADH ratio in C2C12 myoblasts (Figure 6B). The image of immunofluorescence showed that α‐asarone protected mitochondrial mass against KYN challenge (Figure 6C). As same as the role of α‐asarone, ROS scavenger NAC and NAD^+^ precursor NMN also effectively protected mitochondria.

The MAS is a biochemical shuttle to transfer glycolysis‐derived electron across the inner membrane of mitochondria for oxidation, and Slc25a11 and Slc25a12 are the carriers mediating MAS in the exchange of glutamate and ɑ‐ketoglutarate (ɑ‐KG) (Figure 6D). Nuclear respiratory factor‐1 (NRF1) is a transcriptional activator that encodes the mitochondrial genome in cooperation with PGC‐1α.^36^ KYN also impaired the gene expression of *Nrf1*, *Slc25a11*, and *Slc25a12*, which were normalized by α‐asarone and NMN (Figure 6E). NRF1 activates genes encoding factors that mediate replication and transcription of the mitochondrial genome. α‐Asarone protected gene induction of *Kyat1*, *Kyat3*, *Kyat4*, *Slc25a11*, *Slc25a12* and respiratory chain protein (*Cyt b*, *Cyt c*, *Atp5b* and *Cox2*) in a manner dependent on *Nrf1* induction (Figure 6F, Figure S4), suggesting that α‐asarone preserved NAD^+^ to improve KAT/MAS integration through mitochondrial biogenesis. The reduced level of KYN and increased level of KYNA by α‐asarone were diminished using MAS inhibitor AOA in KYN‐treated myoblasts (Figure 6G). PGC‐1α activator ZLN005 reduced and increased KYN and KYNA contents, respectively, but these effects were diminished by si*Slc25a11* or si*Slc25a12* knockdown (Figure 6H). These results indicated that KYN metabolism coupled the MAS function in the context of NAD^+^ renewal and mitochondrial biogenesis.^37^

### 3.7 PGC‐1ɑ is required for α‐asarone to promote KYN disposal in muscle

**FIGURE 7:**
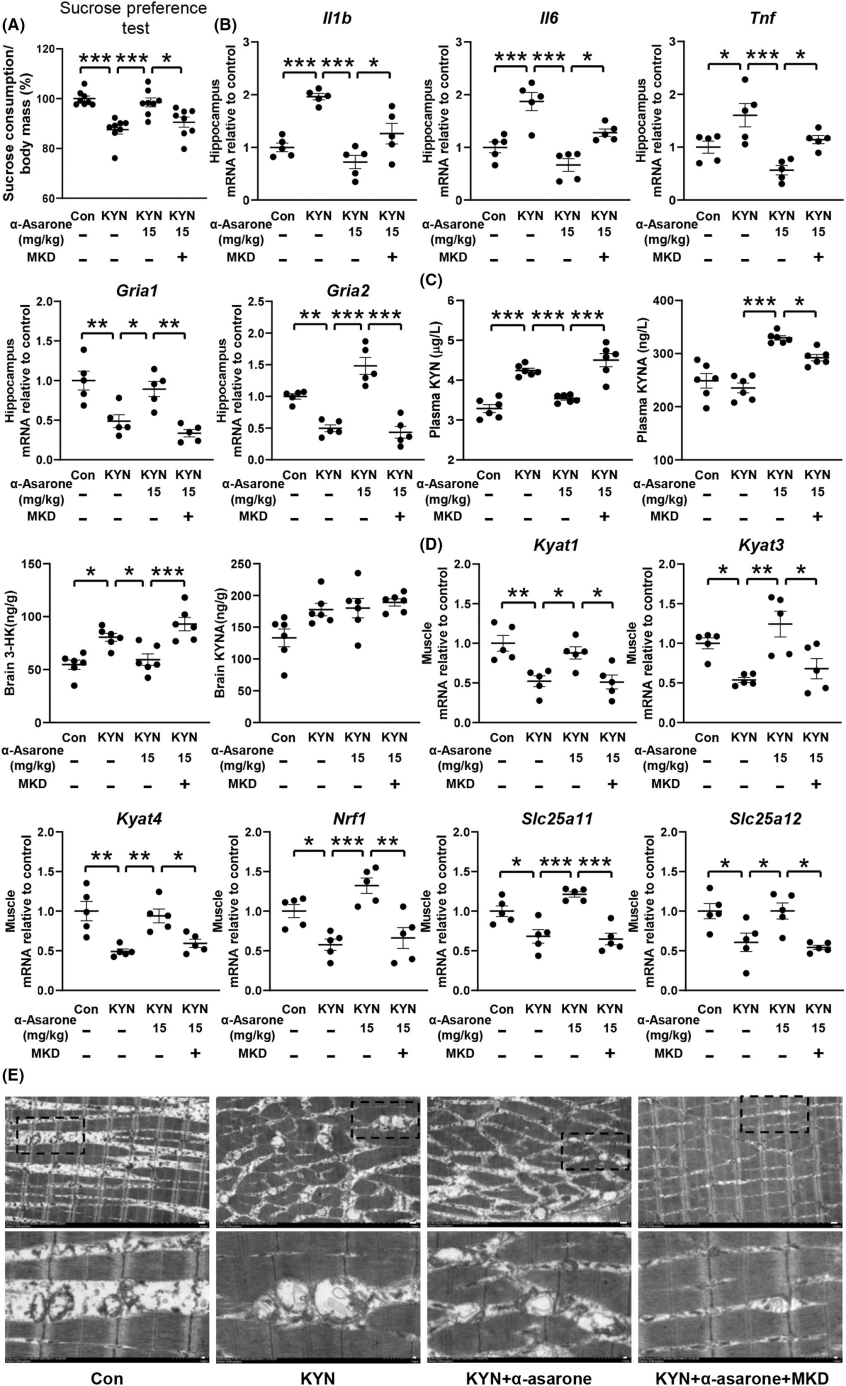
PGC‐1ɑ is required for α‐asarone to promote KYN disposal in muscle. (A) Sucrose preference test (*n* = 8). (B) Gene expression of AMPA receptor subunits and proinflammatory cytokines in hippocampus (*n* = 5). (C) Plasma and brain concentrations of KYN, 3‐HK, and KYNA (*n* = 6). (D) Gene expression of *Kyat1*, *Kyat3*, *Kyat4*, *Nrf1*, *Slc25a11*, *Slc25a12* in gastrocnemius muscle (*n* = 5). (E) Mitochondrial morphology (*n* = 5) in gastrocnemius muscle, scale bar = 5 μm or 1 μm. (KYN, 2.5 mg/kg; α‐asarone, 15 mg/kg; muscle‐specific PGC‐1α knockdown, MKD). Data are expressed as mean ± SEM, *p < 0.05, **p < 0.01, ***p < 0.001 compared with KYN.

To further confirm PGC‐1α‐dependent role of α‐asarone in vivo, muscle‐specific PGC‐1α was knocked down by gastrocnemius injection of AAV9‐sh*Ppargc1α* in mice. The level of *Ppargc1α* was markedly reduced and α‐asarone induction of *Ppargc1α* was blocked by muscle knockdown of *Ppargc1α*, confirming the efficiency of PGC‐1α depletion (Figure S5). We examined the effect of α‐asarone after KYN challenge. The data of sucrose preference test showed that the antidepressant effect of α‐asarone was lost in PGC‐1α‐deficient muscle (Figure 7A). α‐Asarone reciprocally regulated genes encoding proinflammatory cytokines and glutamate receptors in mouse hippocampus in a manner dependent on muscle PGC‐1α (Figure 7B). Meanwhile, α‐asarone promoted KYN clearance dependently on PGC‐1α (Figure 7C). Concordantly, the promotion effect of α‐asarone on gene expression of *Kyat1*, *Kyat3*, *Kyat4*, *Nrf1*, *Slc25a11*, *Slc25a12* and respiratory chain protein (*Cyt b*, *Cyt c*, *Atp5b*, and *Cox2*) were also attenuated in PGC‐1α deficient muscle (Figure 7D, Figure S6). Compared to the control, mitochondrial number and area were all significantly lower in KYN‐treated muscle, but rescued by α‐asarone dependently on PGC‐1α (Figure 7E, Figure S7). Collectively, these results reproduced the findings in vitro and confirmed that α‐asarone‐regulated PGC‐1α induction to promote KYN disposal in skeletal muscle.

## 4 DISCUSSION

**FIGURE 8:**
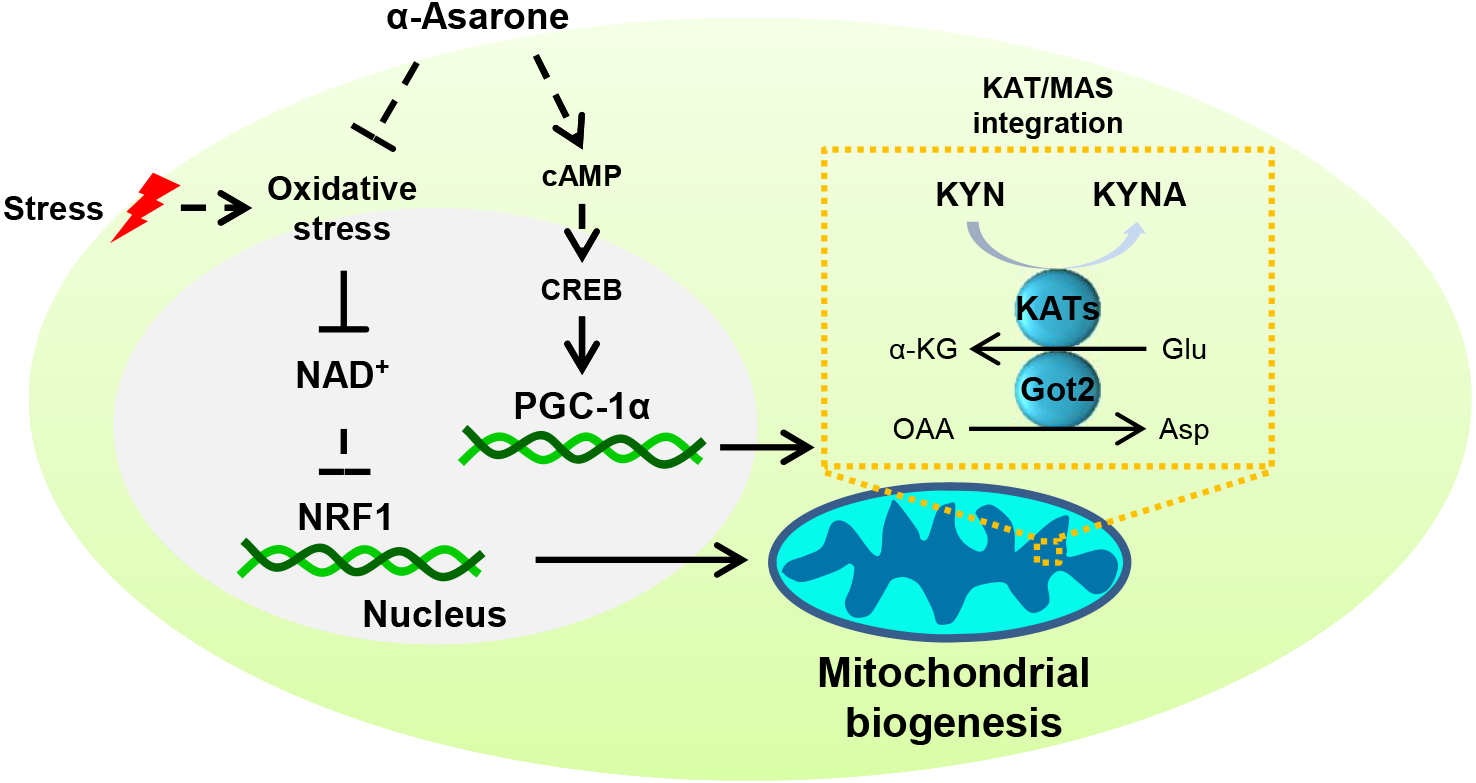
Schematic regulatory mechanism of α‐asarone action. α‐Asarone combats oxidative stress and modulates PGC‐1α induction via cAMP/CREB signaling to promote kynurenine disposal in muscle and thus mediates resilience to depression. (glutamate, Glu; ɑ‐ketoglutarate, ɑ‐KG; oxaloacetate, OAA; aspartate, Asp; glutamic‐oxaloacetic transaminase 2, Got2).

Apart from protein synthesis, most of tryptophan is oxidized along the KYN pathway in the peripheral tissues, whereas KYN accumulation in the brain is neurotoxic, as KYN and downstream metabolites impair neurotransmission and synaptic plasticity.^11^, ^38^ It is reported that α‐asarone alleviated neuronal excitoxicity via GABA_A_ receptors in aged rats and exerted antidepressant effect via noradrenergic and serotonergic receptors in mice.^18^, ^19^ Differently, herein we demonstrated that α‐asarone, by PGC‐1α induction, shifted KYN degradation metabolism toward KYNA shunt in muscle to prevent neurotoxicity, providing new insight into the role of α‐asarone in neuroprotection from the aspect of peripheral intervention (the diagrammatic summary is listed as Figure 8).

In mammals, the majority of free tryptophan is degraded through the KYN pathway, and the metabolites generated are involved in immune response and excitatory neurotransmission. Once crossing the BBB, KYN is further metabolized in astrocytes or microglia, exerting more direct deleterious effects associated with oxidative stress and cell injury.^31^ KYN and 3‐HK accumulation evoke NF‐кB inflammatory signaling and ROS‐mediated neuronal death.^39^, ^40^ In mouse model, CMS induced abnormality in glutamate transmission and synaptic plasticity with inflammation in the brain, and these alterations were accompanied with an increase in 3‐HK contents, which is a downstream metabolite from KYN. In response to stress, tryptophan degradation is initiated in peripheral tissues,^10^, ^11^ and IDOs are activated in the immune system and mucosal tissues such as gut to mediate the conversion of tryptophan to KYN. α‐Asarone reduced 3‐HK contents in the brain, largely due to its role to limit peripheral KYN generation, because circulating KYN is capable of entering the brain to generate 3‐HK. α‐Asarone is shown to act as a GABA_A_ receptor modulator to protect against glutamate toxicity as well as a noradrenergic and serotonergic receptor modulator against depression.^18^, ^19^ Importantly, we found that peripheral regulation of KYN metabolism is a means for α‐asarone to attenuate stress‐induced neural injury.

α‐Asarone reduced peripheral KYN accumulation without affecting the circulating levels of tryptophan, indicating that it specially regulated KYN pathway. Skeletal muscle has recently been found to contribute KYN metabolism.^13^ This regulation happens during exercise training and depends on PGC‐1α induction of KATs to promote KYN to KYNA conversion. Different from KYN and 3‐HK, KYNA is unable to cross the BBB, thereby preventing KYN and 3‐HK accumulation in the brain. Consistent with the action in CMS‐induced depression model, we further confirmed that α‐asarone increased PGC‐1α induction via cAMP/CREB signaling cascades. In fact, skeletal muscle PGC‐1α is highly responsive to cAMP and CREB activation responding to exercise.^41^ cAMP‐mediated PGC‐1α/CREB interaction triggers fibronectin type III domain‐containing protein 5 (FNDC5) expression to shape the metabolic phenotype of myotubes.^42^ cAMP is a second messenger and the implication of signal transduction in mood disorders has been documented,^43^ and our work provides novel insight into the peripheral metabolism.

Muscle PGC‐1α, in cooperation with peroxisome proliferators‐activated receptors α/δ (PPARα/δ), increases KAT expression to reroute KYN pathway for KYNA formation.^13^ This finding also indicates KYN metabolism is linked with mitochondrial function because KAT/MAS integration promotes KYN transamination to replenish glutamate and aspartate pool for energy metabolism.^15^ The MAS transfers reducing equivalents from the cytosol into the mitochondria, and NADH generated is required for mitochondrial complex I to increase respiration rate and maintain mitochondrial membrane potential. We showed that α‐asarone promoted KYN disposal and the MAS function dependently on MAS because the shuttle relies on the reaction of aminotransferase. Interestingly, we found that excessive KYN is also toxic to muscle because it induced oxidative stress to impair mitochondrial structural and functional integrity function. KYN metabolism is involved in inflammatory diseases,^31^ and we showed that uncoupling of KYN metabolism from MAS was an important cause for KYN accumulation in the context of ROS‐associated mitochondrial dysfunction. α‐Asarone exerts the ability to enhance oxidative defense.^44^ When KYN challenge affected MAS by consuming NAD^+^, α‐asarone preserved NAD^+^ to ensure the MAS function. PGC‐1α is a master regulator of mitochondrial biogenesis, responsible for the induction of mitochondrial components including KATs. In combination with other transcriptional factors, PGC‐1α promotes mitochondrial biogenesis, responsible for the induction of mitochondrial components including KATs. In this context, we reasoned that PGC‐1α induction and mitochondrial biogenesis had a contribution to the functional integration of KYN metabolism and MAS. In support, PGC‐1α was shown to protect the heart against endotoxin injury via mitochondrial biogenesis.^45^

To address the role of PGC‐1α, we observed the effects of α‐asarone in muscle‐specific PGC‐1α‐deficient mice and further confirmed that α‐asarone promoted KYN disposal to prevent KYN accumulation, providing evidence in vivo to support the finding observed in vitro. However, we should note that the role of α‐asarone demonstrated here is not the only reason for its anti‐depression action. In fact, α‐asarone is a multifunctional component and more pathological factors are involved in the development of depression. In addition, although promotion of KYN disposal in periphery plays a critical role in neuroprotection, the potent implication on KYN metabolism in astrocytes or microglia is an important subject remained to be revealed. Therefore, a comprehensive study considering systemic regulation from different aspects is needed for the full understanding of the antidepressant role of α‐asarone.

## 5 CONCLUSIONS

Overall, we concluded that KATs coupled with MAS to promote KYN disposal in muscle. α‐Asarone regulated PGC‐1α through cAMP/CREB signaling cascades and protected mitochondrial integrity against oxidative stress to ensure KYN clearance. These results address that protection of muscle mitochondria is an important means for pharmacological intervention to prevent KYN neurotoxicity in the context of stress‐induced depression.

## AUTHOR CONTRIBUTIONS

LY and BL designed the project. LY drafted the manuscript. BL revised the manuscript. LY and CL performed the experiments. LX, YQ, PS and MW contributed to the experiments, data collection, and discussion.

## FUNDING INFORMATION

This study was supported by grants from the National Natural Science Foundation of China (81803758 to LY and 81603374 to MW), Natural Science Foundation of Jiangsu Province (BK20200296 to YQ), the China Postdoctoral Science Foundation (2022 M712812 to LY), and the Project for Hangzhou Medical Disciplines of Excellence and Key Project for Hangzhou Medical Disciplines.

## CONFLICT OF INTEREST

The authors declare no competing financial interests.

## PERMISSION TO REPRODUCE MATERIAL FROM OTHER SOURCES

This is an open access article under the terms of the Creative Commons Attribution License, which permits use, distribution, and reproduction in any medium, provided the original work is properly cited.

## Supporting information


Figure S1

Figure S2

Figure S3

Figure S4

Figure S5

Figure S6

Figure S7

Table S1

## Data Availability

All data supporting the findings of this study are available from the corresponding authors upon reasonable request.
