# Supplementary material for: Alpha‐Asarone modulates kynurenine disposal in muscle and mediates resilience to stress‐induced depression via PGC‐1α induction
Source: CNS Neurosci Ther. 2022 Dec 27;29(3):941–56. doi: 10.1111/cns.14030 (PMC9928554; doi:10.1111/cns.14030)
Supplement: Supplementary file 1 — Figure S1 [file CNS-29-941-s007.docx]

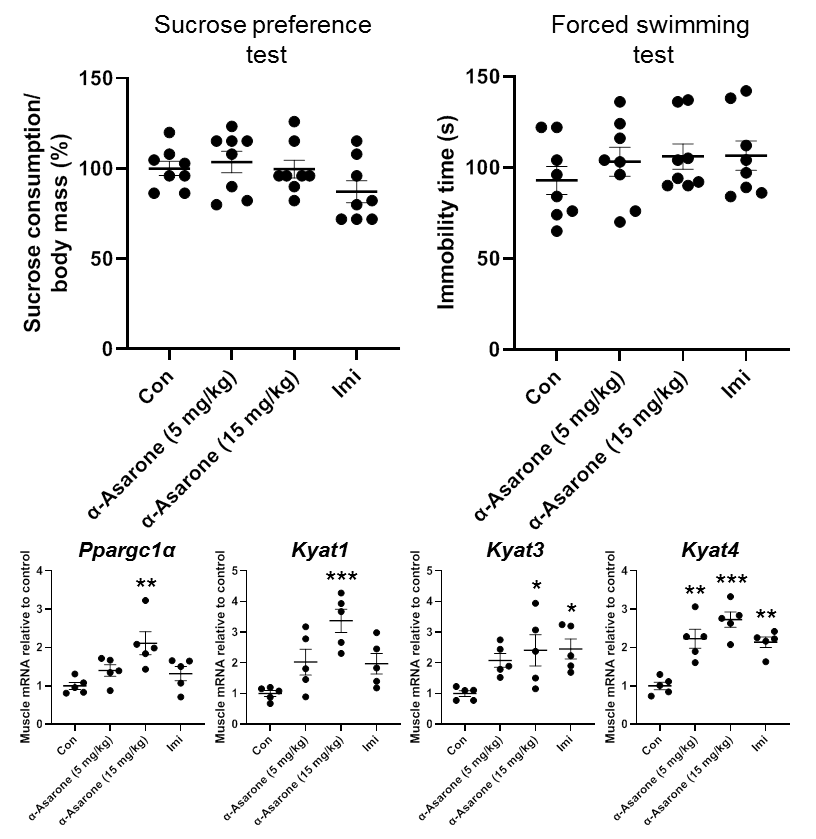


Figure S1 Effect of α-asarone on normal mice. Sucrose preference and forced swimming tests (*n* = 8). Gene expression of *Ppargc1α*, *Kyat1*, *Kyat3*, and *Kyat4* in gastrocnemius muscle (*n* = 5). (Imi, 30 mg/kg). Data are expressed as mean ± SEM, **p* < 0.05, ***p* < 0.01, ****p* < 0.001 compared with Con.
