# Supplementary material for: Alpha‐Asarone modulates kynurenine disposal in muscle and mediates resilience to stress‐induced depression via PGC‐1α induction
Source: CNS Neurosci Ther. 2022 Dec 27;29(3):941–56. doi: 10.1111/cns.14030 (PMC9928554; doi:10.1111/cns.14030)
Supplement: Supplementary file 2 — Figure S2 [file CNS-29-941-s004.docx]

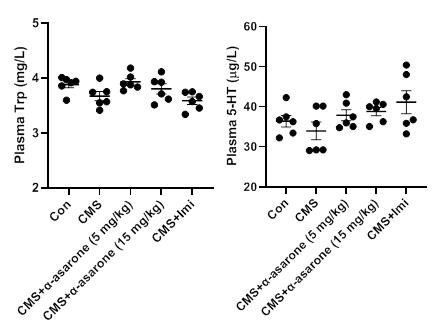


Figure S2 Plasma tryptophan and serotonin concentrations in mice (*n* = 6). (tryptophan, Trp; serotonin, 5-HT; chronic mild stress, CMS; Imi, 30 mg/kg). Data are expressed as mean ± SEM.
