# Supplementary material for: Alpha‐Asarone modulates kynurenine disposal in muscle and mediates resilience to stress‐induced depression via PGC‐1α induction
Source: CNS Neurosci Ther. 2022 Dec 27;29(3):941–56. doi: 10.1111/cns.14030 (PMC9928554; doi:10.1111/cns.14030)
Supplement: Supplementary file 3 — Figure S3 [file CNS-29-941-s005.docx]

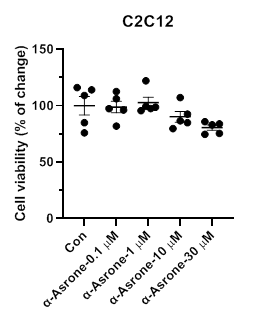


Figure S3 Effect of α-asarone on the growth of myoblasts. Cells were treated with α-asarone for 24 h. A cell viability and proliferation test (using the colorimetric MTT assay) was performed (*n* = 5). Data are expressed as mean ± SEM.
