# Supplementary material for: Alpha‐Asarone modulates kynurenine disposal in muscle and mediates resilience to stress‐induced depression via PGC‐1α induction
Source: CNS Neurosci Ther. 2022 Dec 27;29(3):941–56. doi: 10.1111/cns.14030 (PMC9928554; doi:10.1111/cns.14030)
Supplement: Supplementary file 5 — Figure S5 [file CNS-29-941-s001.docx]

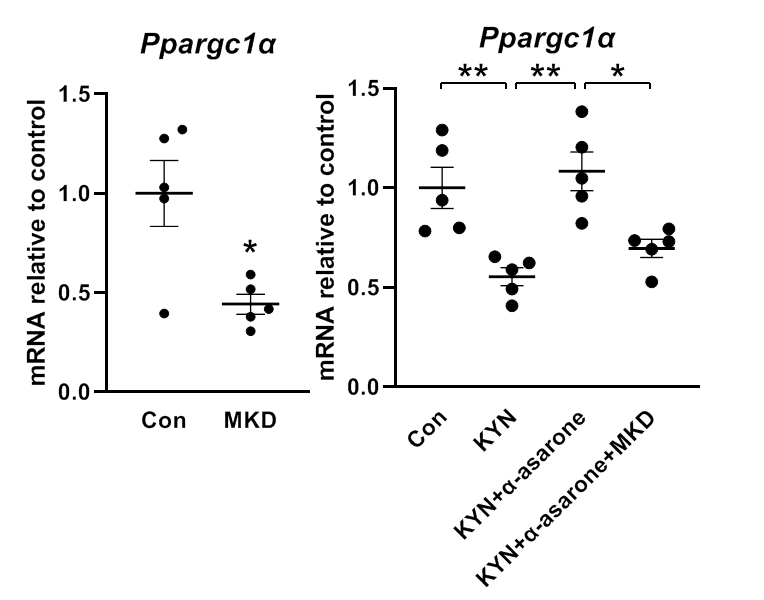


Figure S5 *Ppargc1α* mRNA level in muscle-specific PGC-1α knockdown mice. Gene expression of *Ppargc1α* in muscle (*n* = 5). (α-asarone, 15 mg/kg; KYN, 2.5 mg/kg; muscle-specific PGC-1α knockdown, MKD). Data are expressed as mean ± SEM. **p* < 0.05, ***p* < 0.01.
