# Supplementary material for: Alpha‐Asarone modulates kynurenine disposal in muscle and mediates resilience to stress‐induced depression via PGC‐1α induction
Source: CNS Neurosci Ther. 2022 Dec 27;29(3):941–56. doi: 10.1111/cns.14030 (PMC9928554; doi:10.1111/cns.14030)
Supplement: Supplementary file 6 — Figure S6 [file CNS-29-941-s008.docx]

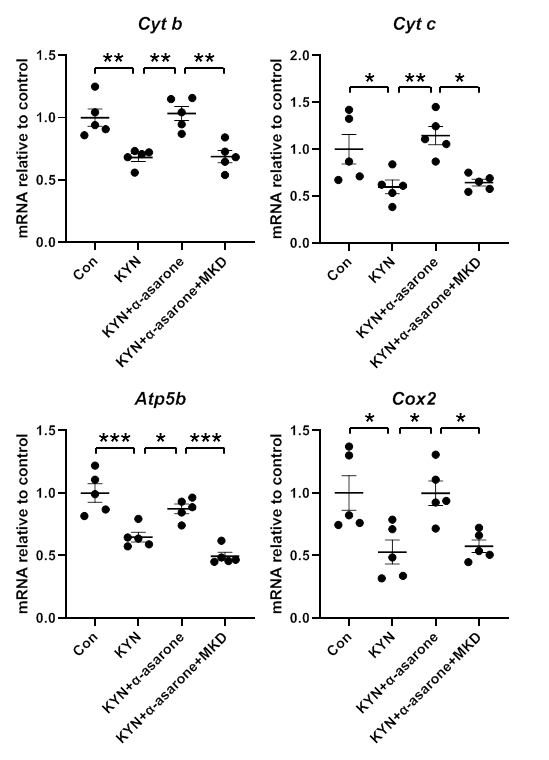


Figure S6 α-Asarone regulates the expression of mitochondrial respiratory chain protein in mice. Gene expression of *Cyt b*, *Cyt c*, *Atp5b* and *Cox2* in gastrocnemius muscle (*n* = 5). (KYN, 2.5 mg/kg; α-asarone, 15 mg/kg; muscle-specific PGC-1α knockdown, MKD). Data are expressed as mean ± SEM, **p* < 0.05, ***p* < 0.01, ****p* < 0.001 compared with KYN.
