# Supplementary material for: Alpha‐Asarone modulates kynurenine disposal in muscle and mediates resilience to stress‐induced depression via PGC‐1α induction
Source: CNS Neurosci Ther. 2022 Dec 27;29(3):941–56. doi: 10.1111/cns.14030 (PMC9928554; doi:10.1111/cns.14030)
Supplement: Supplementary file 7 — Figure S7 [file CNS-29-941-s006.docx]

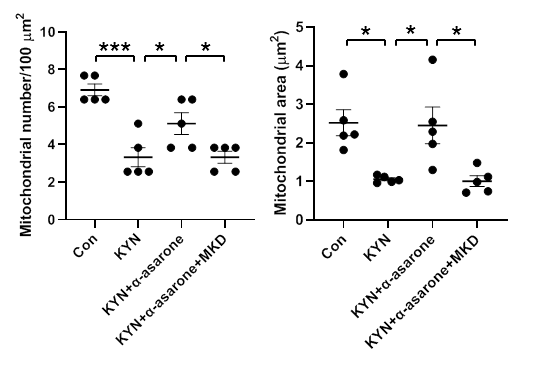


Figure S7 Mitochondrial number and area in muscle-specific PGC-1α knockdown mice. Mitochondrial number and area (*n* = 5) in gastrocnemius muscle, scale bar = 5 μm or 1 μm. (KYN, 2.5 mg/kg; α-asarone, 15 mg/kg; muscle-specific PGC-1α knockdown, MKD). Data are expressed as mean ± SEM, **p* < 0.05, ***p* < 0.01, ****p* < 0.001 compared with KYN.
